# HIV-1 transmission networks in high risk fishing communities on the shores of Lake Victoria in Uganda: A phylogenetic and epidemiological approach

**DOI:** 10.1371/journal.pone.0185818

**Published:** 2017-10-12

**Authors:** Sylvia Kiwuwa-Muyingo, Jamirah Nazziwa, Deogratius Ssemwanga, Pauliina Ilmonen, Harr Njai, Nicaise Ndembi, Chris Parry, Paul Kato Kitandwe, Asiki Gershim, Juliet Mpendo, Leslie Neilsen, Janet Seeley, Heikki Seppälä, Fred Lyagoba, Anatoli Kamali, Pontiano Kaleebu

**Affiliations:** 1 Medical Research Council/Uganda Virus Research Institute, Research Unit on AIDS, Entebbe, Uganda; 2 Aalto University, School of Science, Department of Mathematics and Systems Analysis, Espoo, Finland; 3 UVRI/IAVI HIV Vaccine Program, Entebbe, Uganda; 4 International AIDS Vaccine Initiative, New York, United States of America; 5 London School of Hygiene and Tropical Medicine, London, United Kingdom; Institut Pasteur of Shanghai Chinese Academy of Sciences, CHINA

## Abstract

**Background:**

Fishing communities around Lake Victoria in sub-Saharan Africa have been characterised as a population at high risk of HIV-infection.

**Methods:**

Using data from a cohort of HIV-positive individuals aged 13–49 years, enrolled from 5 fishing communities on Lake Victoria between 2009–2011, we sought to identify factors contributing to the epidemic and to understand the underlying structure of HIV transmission networks. Clinical and socio-demographic data were combined with HIV-1 phylogenetic analyses. HIV-1 *gag*-p24 and *env*-gp-41 sub-genomic fragments were amplified and sequenced from 283 HIV-1-infected participants. Phylogenetic clusters with ≥2 highly related sequences were defined as transmission clusters. Logistic regression models were used to determine factors associated with clustering.

**Results:**

Altogether, 24% (n = 67/283) of HIV positive individuals with sequences fell within 34 phylogenetically distinct clusters in at least one gene region (either *gag* or *env*). Of these, 83% occurred either within households or within community; 8/34 (24%) occurred within household partnerships, and 20/34 (59%) within community. 7/12 couples (58%) within households clustered together. Individuals in clusters with potential recent transmission (11/34) were more likely to be younger 71% (15/21) versus 46% (21/46) in un-clustered individuals and had recently become resident in the community 67% (14/21) vs 48% (22/46). Four of 11 (36%) potential transmission clusters included incident-incident transmissions. Independently, clustering was less likely in HIV subtype D (adjusted Odds Ratio, aOR = 0.51 [95% CI 0.26–1.00]) than A and more likely in those living with an HIV-infected individual in the household (aOR = 6.30 [95% CI 3.40–11.68]).

**Conclusions:**

A large proportion of HIV sexual transmissions occur within house-holds and within communities even in this key mobile population. The findings suggest localized HIV transmissions and hence a potential benefit for the test and treat approach even at a community level, coupled with intensified HIV counselling to identify early infections.

## Introduction

Global health initiatives have led to a reduction in HIV transmission at population level [1]. However, sub-Saharan Africa still accounts for 70% of the global total of new HIV infections [2]. Antiretroviral therapy (ART) remains vital to the management of infection in the absence of a cure or a vaccine.

Fishing communities have been characterised as among populations that are at high risk for HIV infection in sub-Saharan Africa and South-east Asia despite prevention efforts [3, 4]. New HIV infections continue to occur in Uganda despite the country's wide roll out of HIV-related services and other interventions [5–8]. As in other sub-Saharan countries, the HIV epidemic is characterised by significant disparities in age, sex and geographical areas [9]. Fishing communities in Uganda represent a growing proportion of the HIV epidemic with HIV incidence rates over 4 times higher and prevalence at least 3 times higher compared to the general population [10–12].

Efforts to reach minority groups with high HIV infection rates, particularly "mobile" fisherfolk, have not been successful in the past [4, 13]. New approaches combining phylogenetic with epidemiological data have advanced our knowledge of sexual networks, and combined with statistical methods may provide insights into underlying risk factors for local HIV epidemics [14, 15]. Few studies incorporating phylogenetics have been conducted among populations with predominant HIV A/D subtype infections, which occur mainly in East Africa to understand complex HIV transmission dynamics at a population level [16–18]. A thorough understanding of drivers of individual epidemics is required to develop, implement and maintain effective HIV prevention programmes [15, 19, 20].

Our main aim was to identify factors contributing to the on-going HIV epidemic among fisherfolk in Uganda, combining clinical and socio-demographic data with HIV-1 phylogenetic analyses from HIV *gag* and *env* gene regions. First, we describe temporal and biological dynamics, potentially unmasking important factors that drive the epidemic, which may otherwise not be seen in the patients' clinical and epidemiological profile. Secondly, we characterise the composition of the phylogenetic clusters and specifically assess where the potential transmissions occurred, investigating factors associated with cluster membership.

## Methods

### Study population: Fishing communities

**Fig 1:**
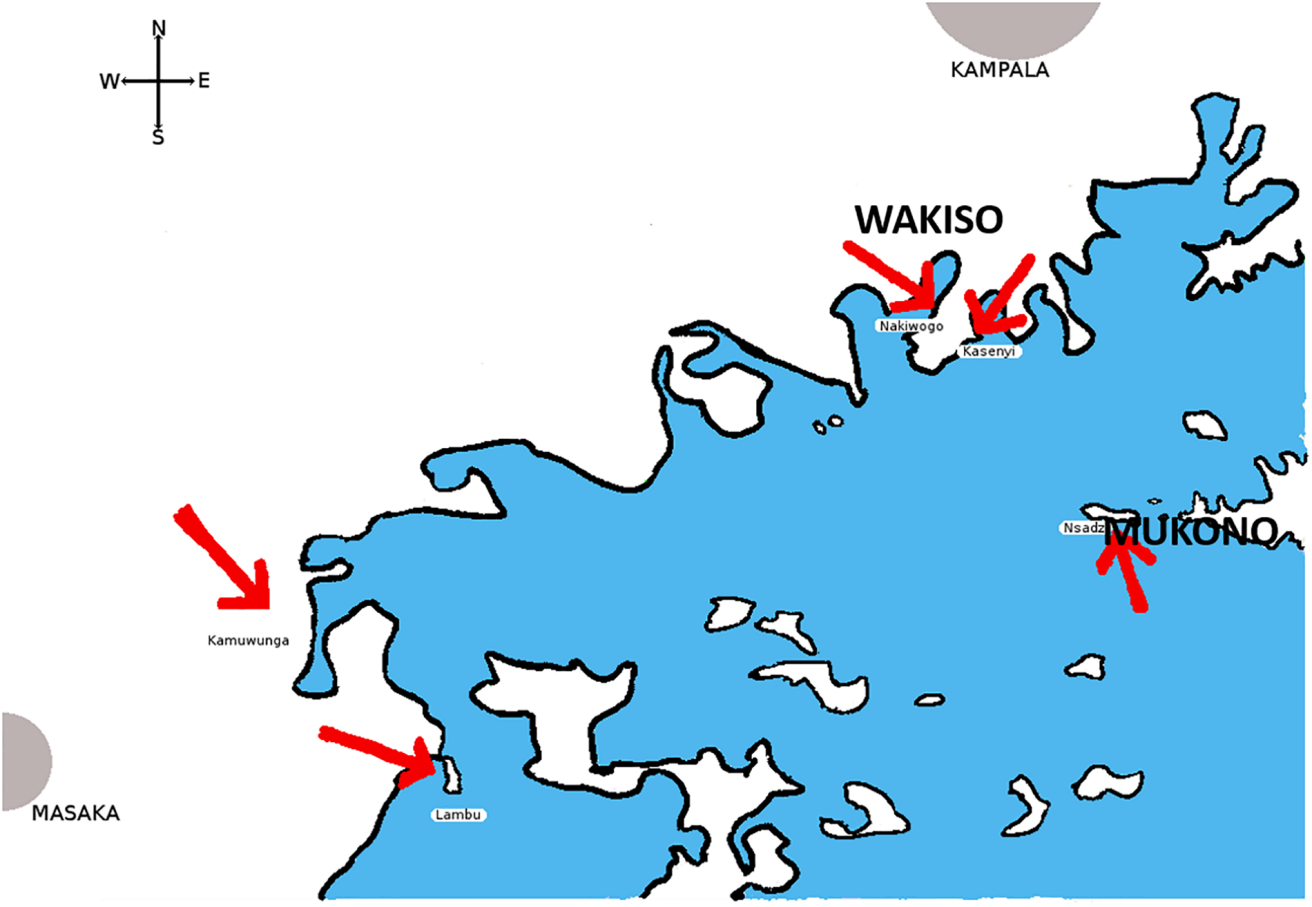
Study area. Lake shore districts Masaka, Mukono and Wakiso.

Between February and August 2009, 2074 individuals aged 13–49 across 5 fishing communities from 3 lakeshore districts (Masaka, Wakiso and Mukono) in Uganda were screened for enrolment. The three lake shore districts are shown in Fig 1. Two thirds (10188/15415) of the total population within the 5 sites were aged 13–49, and 2074 represents 20% (2074/10188) of the individuals aged 13–49 years. Eligible persons in this study were defined by age (13–49), residence (able and willing to provide locator information), willing to undergo HIV testing, pregnancy test if female, willing to be interviewed and interest in study. Exclusion criteria included participation in another study, presence of any condition that would interfere with study objectives. Eligible consenting adults defined as sexually active and at risk of HIV infection were enrolled. At screening, all consenting individuals were tested for HIV using a Determine HIV-1/2 rapid test (Abbot Laboratories, Diagnostic division, Chicago, IL, USA) and 2 independent ELISA tests for confirmation.[10] Participants identified as HIV-infected at screening were referred to as HIV prevalent. In this study, we analysed data from HIV prevalent and HIV incident individuals.

**Fig 2:**
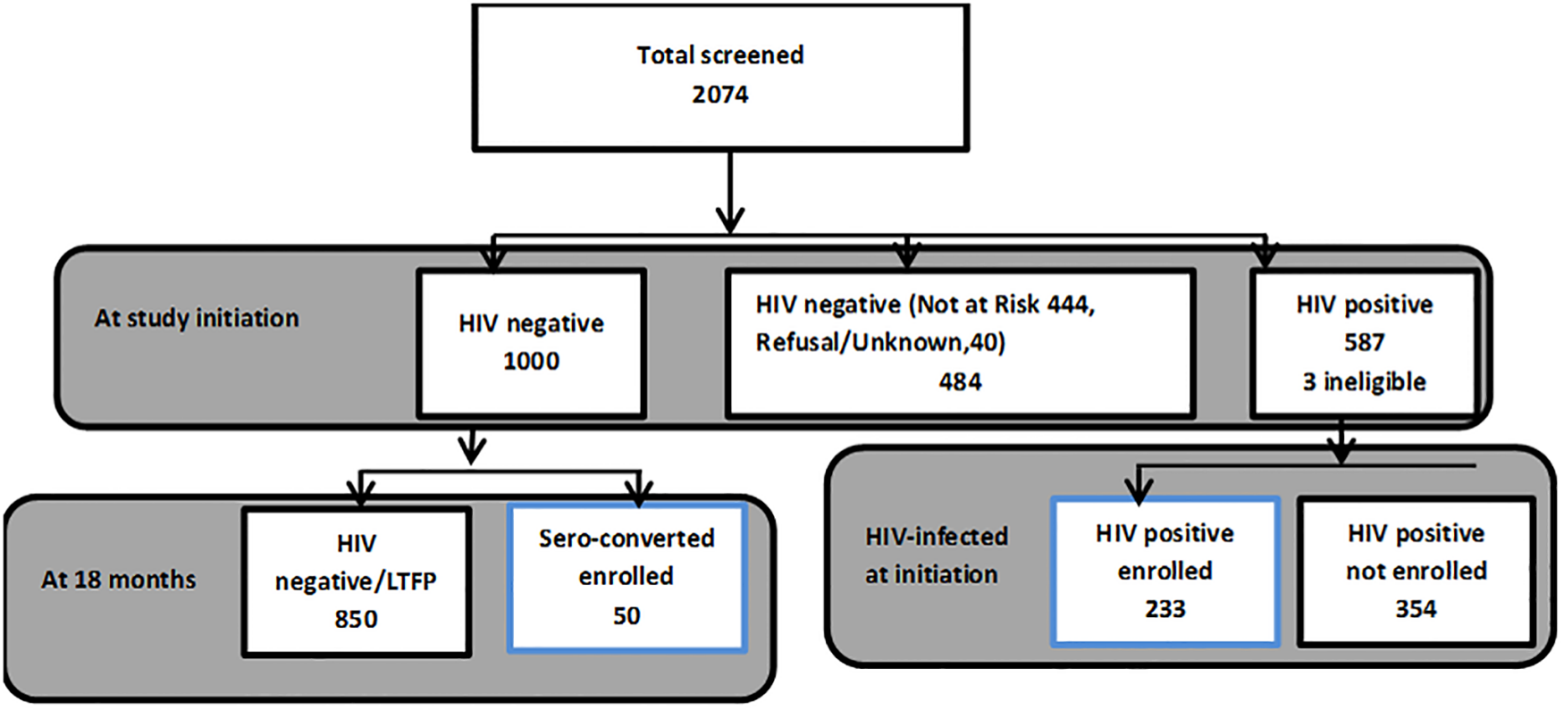
Enrolment of study participants.

Of the 2074 screened individuals, approximately 67% (1000/1484) of all HIV uninfected individuals aged 13–49 years were enrolled in a sub-study and followed up 6-monthly for 18 months (see Fig 2). Within the present study, we analysed data for all incident cases identified during follow-up of the 1000 individuals within the previous sub-study to assess HIV incidence and risk behavior.[12] We also planned to enroll 250 (42%) HIV prevalent individuals selected sequentially (100 from Masaka and 100 from Wakiso and an additional 50 from Wakiso). Both HIV prevalent and incident individuals have previously been described in detail [10, 12]. A household in our study is defined as a group of individuals who cook, eat and live together, and a village is the lowest political administrative unit in Uganda. A village/community may consist of 50–70 households and is governed by a Local Council (LC) I, and at the LC II level is a parish. At the next level are sub-counties followed by several counties which make up the district.

At each clinic visit, data on socio-demographic variables such as residence, marital status, education, occupation, travel history, reported partnerships and sexual behaviour were collected using questionnaires in face to face interviews. Clinical data on CD4 cell count, genital sores/discharge, and syphilis infection was obtained at each visit [10]. We excluded 3 individuals that were not eligible. The majority of HIV-infected individuals were not on ART. All individuals identified as HIV-infected were referred to their preferred HIV/AIDS care provider.

The study was approved by the Uganda Virus Research Institute Research and Ethics Committee and the Uganda National Council for Science and Technology. We obtained written informed consent from individuals aged ≥18 years documented on forms approved by ethics review committees. Written informed consent was independently obtained from emancipated minors (13–17 years) enrolled in the study following national guidelines and approved by the ethics review committees.

### Sample collection, DNA extraction and PCR amplification

HIV-1 sero-positive volunteers donated 10ml of blood (prevalent cases), as did volunteers who sero-converted during follow up (incident cases). Proviral DNA was extracted using the Qiagen DNA isolation kit (Qiagen, Hilden, Germany) from EDTA-treated blood. Partial *gag* encoding 463 bp of the HIV-1 capsid protein p24 was amplified by nested PCR as previously described [21]. Partial *env* gene encoding about 460bp of gp41 was also amplified by nested PCR as previously described [22].

### Sequencing, subtyping and phylogenetic analysis

#### Sequencing

The PCR products were purified using a QIAquick PCR purification kit (Qiagen, Valencia, CA) and sequenced in the sense and antisense direction with the nested *gag* and gp41 primers described above. All sequencing reactions were performed using an in-house assay at the MRC/UVRI sequencing facility using an automated 4-capillary ABI3130 genetic analyser. Nucleotide sequences were assembled and edited using Sequencher version 5.2.4-sequence analysis software (Gene Codes Corporation, Ann Arbor, MI USA)

#### HIV subtyping

We performed subtyping of the generated sequences using the COMET [23] and REGA softwares [24]. Sequences that were unassigned by both softwares were considered unique recombinants.

#### Phylogenetic analysis

Subtype reference sequences of HIV-1 group M from the Los Alamos Sequence database [25], were used to automatically align the generated sequences using ClustalX [26]. The software ViroBLAST was used to scan public databases for sequences similar (bootstrap ≥95%) to our sequences and for checking contamination [27]. We further used ElimDupes software to compare the ViroBLAST, subtype references and the study sequences to eliminate any duplicate or very similar sequences [28]. The sequences obtained using ViroBLAST were used with the subtype reference sequences to construct Maximum Likelihood trees using PhyML Software [29], and the reliability of tree topologies estimated by bootstrap analysis (1000 replicates) [30]. We performed analysis on the two gene datasets to determine the transmission network genetic distance and bootstrap thresholds. We used different genetic distance thresholds (0.5%–5%) and bootstrap of ≥95% to determine the optimal thresholds as described in detail in the supplementary materials. Phylogenetic HIV transmission pairs and clusters (containing ≥ 2 sequences) were defined as those with bootstrap support of ≥95% with a maximum pairwise genetic distance within the transmission pair or cluster of 3.5% for *gag* and 4.5% for *env* using the Cluster Picker v1.3 software [31]. In this study, pairs (n = 2) and larger clusters (n≥3) are all referred to as clusters. For quality control purposes, samples from the identified clusters underwent in-house Quality Assurance checks to rule out cross-contamination. Sequences from this study were deposited in GenBank under accession numbers KX682425—KX682694 and KX682695—KX682974.

### Statistical analysis

Sequences for *gag* and/or *env* regions were obtained for 283 HIV-infected individuals who included the incident cases (see Methods above) and approximately 40% (233/590) HIV prevalents identified at screening. We analysed geographical data as discrete-state ie location as a discrete variable attached to a household. This analysis included villages, households and clusters identified to visualise the sexual networks distribution across the communities/ districts. The distance between households was randomly allocated and may not accurately represent household distribution but serves to visualise characteristics of clusters by location. A heat map was generated to detect salient group structures of clustering (i.e. whether there is more clustering in some particular groups). The proportions in the heat map were generated using pre-defined baseline categories of the variables. A few individuals (n = 5) could not be classified into a heat map category due to missing data. Chi-square tests for independence and logistic regression models were used to investigate factors associated with clustering of the HIV-infected individuals. We only considered one cluster if the cluster was identical in both gene regions. Factors assessed included; age, sex, travel away in the last 3 months, HIV-subtype, self-reported genital sores, alcohol use and as demonstrated from other studies, living with an HIV-infected individual in the same household. We further explored associations with clustering using more stringent and epidemiologically plausible genetic distance thresholds at 1.0% and 1.5%. All statistical analyses were done using R software version 3:2:2.

## Results

### Summary characteristics of the HIV fishing communities cohort

Our study population included 50 HIV-infected incident individuals who sero-converted during follow-up (see Methods) and 233 HIV-infected prevalent individuals. Altogether 283 represented 44% (n = 283/640) of all HIV positive participants. Of the planned 250 HIV prevalent cases identified at screening, 233 were considered representing 40% of all 587 HIV prevalent cases (ie sequenced, n = 233 and unsequenced, n = 354). Of 233 HIV prevalent, 93 were from Masaka, 95 from Wakiso and 45 from Mukono. Reasons for missing sequences were due to insufficient sample.

Overall 45% were men, median (IQR) age was 29(25, 35), 61% were married, 24% widowed/divorced and 13% single and the remainder <1% unknown. 54% had lived within the study area for less than 5 years and 46% reported being away from home in the last 3 months, Table 1.

**Table 1:** Summary of baseline characteristics for the 3 lakeshore districts surveyed in 5 fishing communities between 2009–2011.

Characteristic	All HIV infected	HIV infected Un-sequenced*	HIV individuals sequenced**	p-value
	N = 637	N = 354	N = 283	
Number of Households	281	155	126	
**Location**				
Masaka	394 (62%)	270 (76%)	124 (44%)	< 0:001
Wakiso	170 (27%)	60 (17%)	110 (39%)	
Mukono	73 (1%)	24 (7%)	49 (17%)	
**Sex**: Male	267 (42%)	138 (39%)	129 (45%)	0.06
**Age group**				
13–29	322 (51%)	181 (51%)	141 (49%)	
30–44	297 (47%)	164 (46%)	133 (46%)	
45–50	16 (3%)	9 (3%)	7 (2%)	0.5
Median age (IQR)	29 (25–35)	29 (25–34)	29 (25–35)	
**Marital status**				
Single	102 (16%)	64 (18%)	38 (13%)	
Married	395 (62%)	221 (62%)	174 (61%)	0.1
Divorced/ widowed	138 (22%)	69 (19%)	69 (24%)	
**Duration in community**				
5–45 years	204 (32%)	99 (28%)	105 (37%)	0.01
< 5 years	293 (46%)	139 (39%)	153 (54%)	
Missing information	141 (22%)	116 (33%)	25 (9%)	
**Away in last 3 months**				
Yes vs No	284 (45%)	154 (44%)	130 (46%)	0.7
**Marijuana use**				
Yes vs No	23 (4%)	13 (4%)	10 (4%)	0.6
**Alcohol use**				
Never	247 (39%)	136 (38%)	111 (39%)	
Rarely	167 (26%)	103 (29%)	64 (22%)	0.1
Regularly	202 (31%)	101 (29%)	100 (35%)	

2 individuals missing baseline information were excluded.

*14 individuals missing marijuana use, alcohol use and travel away in last 3 months.

** 8 individuals missing marijuana use, missing alcohol use, travel away in last 3 months and 5 missing marital status.

No differences were observed between those with sequences and without sequences in relation to age, duration of living in the study area, reported genital sores or new partners in the last 3 months (p>0.1) indicating no evidence of bias in the selection of study subjects. We found no difference in marijuana use or alcohol use between sequenced and un-sequenced individuals, suggesting no bias in this and other characteristics associated with risk of HIV-1 transmission. There were differences in the proportions between sequenced and un-sequenced individuals by district/location and gender; 76% in Masaka, 17% in Wakiso, and 7% in Mukono un-sequenced, versus 44%, 39%, and 17% sequenced, respectively, p < 0:001; Of those not sequenced, 39% were men versus 45% in sequenced individuals, p = 0.06.

### HIV subtyping

We found no contaminants or sequences that were very similar to our study sequences when we performed our quality controls using ViroBLAST and ElimDupes softwares. We obtained 542 sequences from 283 HIV-infected individuals who were successfully sequenced in *gag* and/or *env*, Table 2. Most (259/283, 92%) individuals were sequenced in both *gag* and *env* sub-genomic fragments; 71% (183/259) had concordant subtypes and 29% (76/259) had discordant subtypes suggesting infection with intersubtype recombinant viruses in the two regions, whereas 98% (276/283) had sequences only for *gag* and 94% (266/283) in *env* only. Among the 183 that had concordant subtypes in the two genes, 58% (107) were subtype A, 39% (72) were subtype D, 2% (3) were subtype C and <1% (1) were subtype G. Among the 76 that had discordant subtypes in the two genes, the majority (67%, 51) were subtype A and D recombinants, while 33% (25) were other recombinant forms.

**Table 2:** HIV sequences from HIV-infected Individuals in fisherfolk high-risk fishing communities (n = 283).

HIV genotyping		*Gag*- based genotyping						
		A1	C	D	G	URF	NA**	Total
*Env*—based genotyping	A1	107	2	35	0	12	4	160
	B	0	0	2	0	0	0	2
	C	3	3	2	0	0	0	8
	D	16	1	72	0	2	3	94
	G	0	0	0	1	0	0	1
	URF	0	0	1	0	0	0	1
	NA*	7	0	7	2	1	0	17
	Total	133	6	119	3	15	7	283

283 individuals with sequences in *gag* and/or *env* gene regions. URF Unique Recombinant Form sequences;

NA*not amplifiable in *env*;

NA** not amplifiable in *gag*.

Participants in grey had sequences in both gene regions. 65% (183) sequences concordant in *gag* and *env* gene regions.

We found the distribution of HIV-subtypes differed by district/location for concordant subtypes; A (47%), (62%), (79%) and D (53%), (32%), (21%) for Masaka, Wakiso and Mukono respectively (data not shown). Earlier studies in this population showed a predominance of HIV subtype-D in the south west (Masaka district) and subtype-A in the central districts (Wakiso and Mukono). None of the other factors assessed including age group or sex, or new sex partners in last 3 months differed by HIV-subtypes (for concordant subtypes).

### Characteristics of phylogenetic clusters identified from 283 HIV-infected individuals

**Fig 3:**
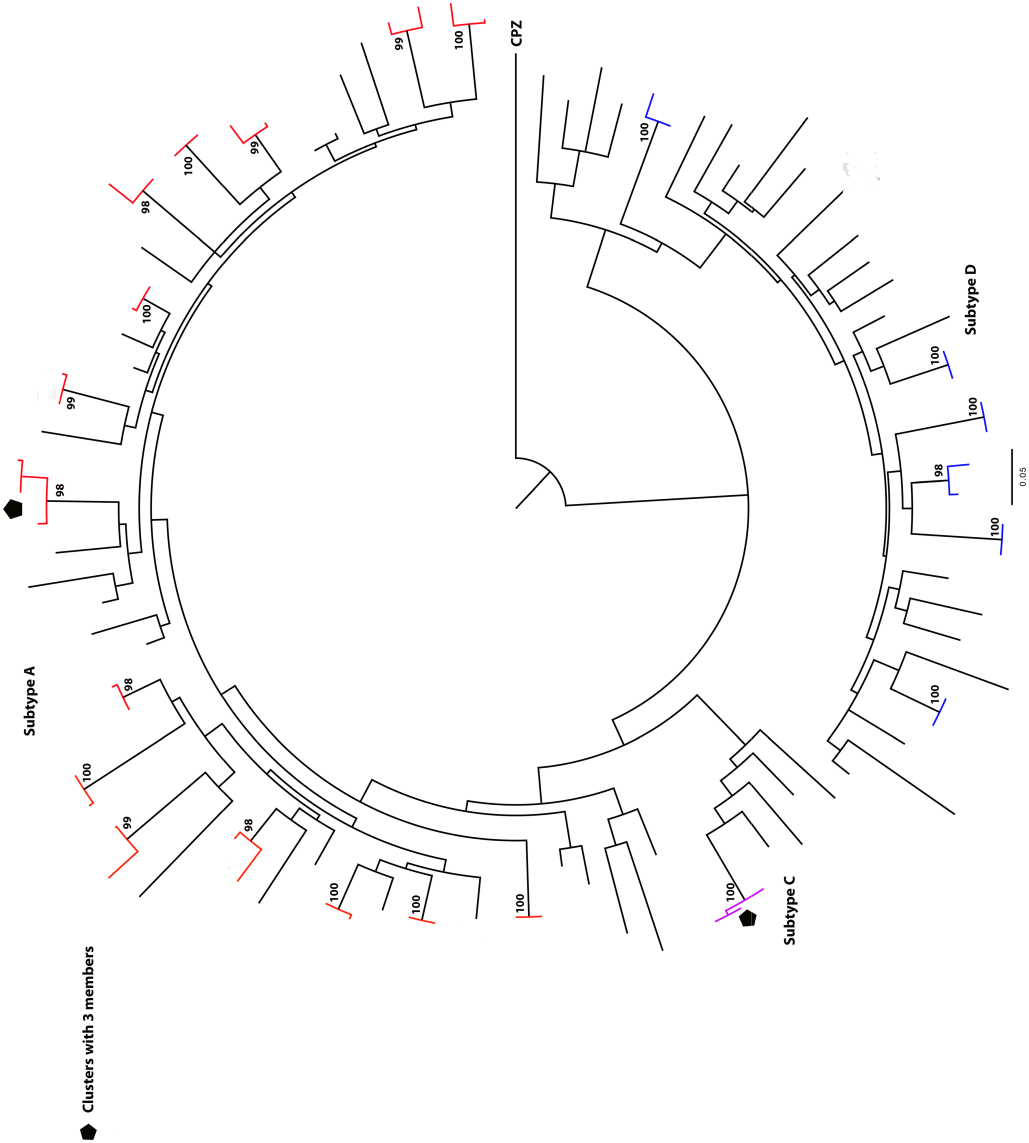
Maximum-likelihood phylogenetic tree showing the HIV transmission clusters in *gag* gene approximately 463 base pairs (n = 25) involving 86 of the 276 participant sequences. Clusters with bootstrap support ≥ 95% and genetic distance of ≤ 3.5% are shown. Three clusters with > 2 members are shown while the rest of clusters had two members. Subtype A clusters are highlighted red and subtype D blue. The tree scale is shown.

**Fig 4:**
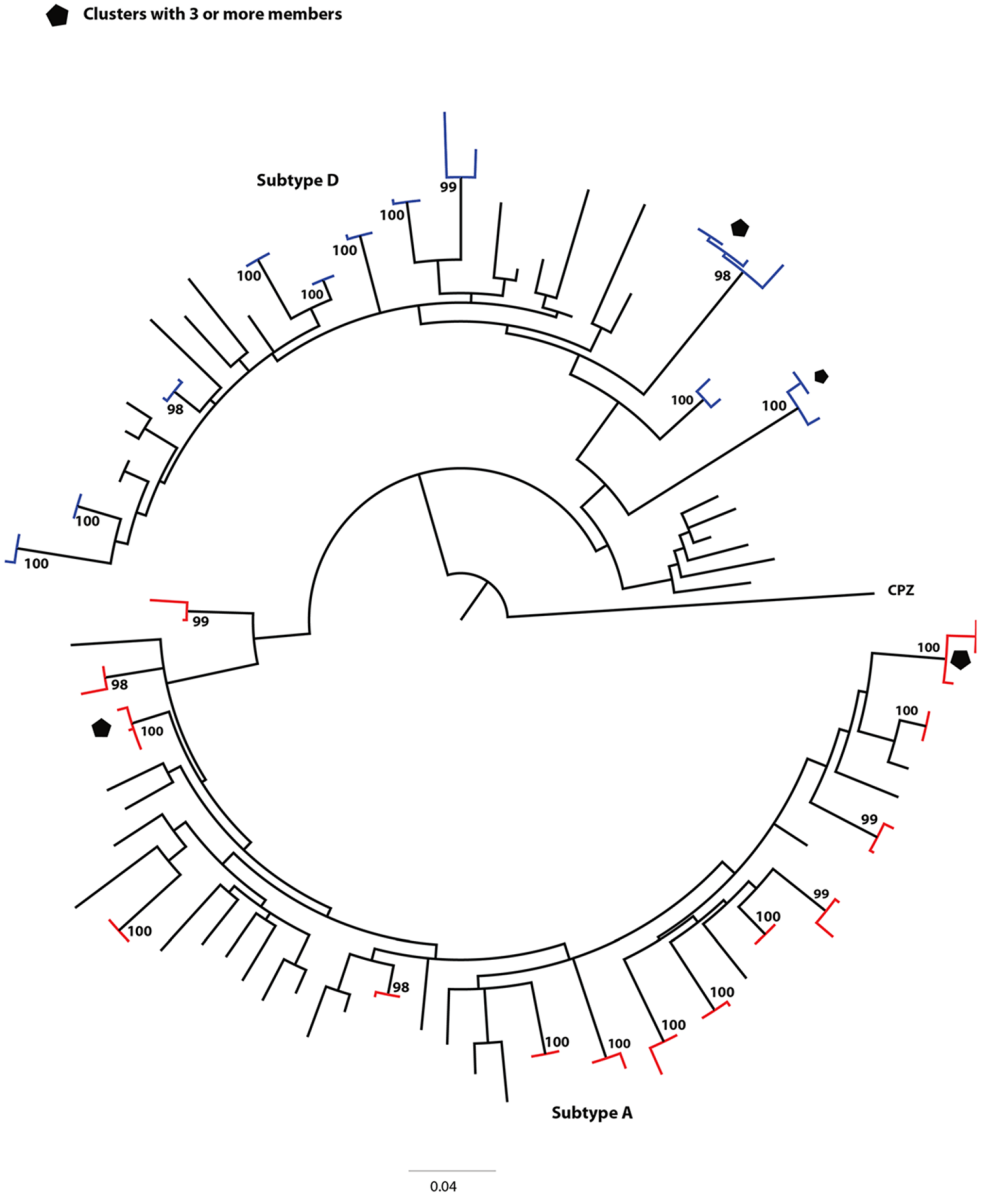
Maximum-likelihood phylogenetic tree showing the HIV transmission clusters in *env* gene approximately 460 base pairs (n = 22) involving 79 of the 266 participant sequences shown while the rest of clusters had two members. One cluster with bootstrap of 99% and genetic distance of ≤ 4.5% is included since it clustered in *gag*. Subtype A clusters are highlighted red, subtype C purple and subtype D blue. The tree scale is shown.

The majority of clusters in *gag* and *env* genes were small with a range of 2–4 individuals per cluster. In *gag*, 24 of the 25 clusters identified were eligible for analysis—see Methods. Of the 24 clusters, 20 included two individuals while three clusters included 3 individuals and one cluster had four individuals (Fig 3). There were 14 subtype A and 11 subtype D clusters in *gag*. In *env*, of the 22 clusters identified, 19 were eligible for analysis (see Methods); 17 included two individuals and two clusters included 3 individuals (Fig 4). There were 15 subtype A, one subtype C and 6 subtype D clusters in *env*. One *env* cluster with bootstrap of 99% and genetic distance of 4.7% was included since it clustered in *gag* and was a confirmed sexual partnership. Nine clusters (18 individuals within pair group) were identified in both *gag* and *env* gene trees.

Overall 24% (67/283) individuals were clustered in either *gag* or *env* gene regions. We identified 34 distinct clusters (29 pairs, 4 with 3 individuals and 1 with 4 individuals), majority contained male-female groupings 16/34 (47%), the remaining 38% were male only (6/34, male/male) or female only (7/34, female/female), Table 3. The 4 larger clusters of three each contained 2 females and one male and 1 cluster of 4 contained 2 males and 2 females. At least one incident case was found in 11/34 (32%) clusters (9 within pair and 2 within the 3 individual group) and 4 of 11 were exclusively incident pairs. Among incident cases we found 14/50 (28%) clustered versus 53/233 (23%) prevalent cases, see Table 4. Furthermore, among incident cases, 5/14 (36%) were transmissions linked within household (data not shown).

**Table 3:** Characteristics of 34 clusters identified in *gag* or *env* gene regions for 283 HIV-infected participants 2009–2011.

Cluster char-acteristics	All clusters	Potential transmission clusters (Clusters with at least one incident case)	Clusters with only incidents (includes those in column 2)	Gender distribution
Cluster size	2 (29)	9	4	7 FF
				16 MF
				6 MM
	3 (4)	2	0	4 MFF
	4 (1)	0	0	1MMFF
Within Household*	8 (23.5%)	3	2	1 FF
				7 MF
Within village/ community	20 (58.8%)	7	2	5 FF
				7 MF
				3 MFF
				4 MM
				1 MMFF
Cross Village/ community	3 (8.8%)	1	0	2 MF
				1 MM
Cross district	3 (8.8%)	0	0	1 MM
				1 MFF
				1 FF

*7/8 of these clusters within reported stable sexual partnerships

**Table 4:** Characteristics of sequenced data 34 clusters (n = 283).

	Non-clustered (n = 216) %	Clustered (n = 67*) %	Crude OR (95% CI)	P-value	Adjusted OR (95% CI)	P-value
**Sex**: Male	97 (44)	34 (46)	2.52 (0.49,13.0)			
**Age group**						
13–29	105 (49)	36 (54)	1			
30–44	104 (48)	29 (43)	0.73 (0.42, 1.27)			
45–50	5 (2)	2 (3)	1.03 (0.19, 5.53)	0.5		
Incident	36 (16)	14 (21)				
**Marital status**						
Single	28 (13)	10 (15)	1			
Married	130 (60)	44 (65)	1.13 (0.49, 2.56)			
Divorced,	56 (26)	13 (19)	0.76 (0.29, 1.99)	0.5		
widowed						
**Alcohol use**						
Never	81 (38)	30 (45)	1			
Rarely	50 (23)	14 (21)	0.63 (0.31, 1.28)			
Regularly	80 (37)	20 (30)	0.60 (0.32, 1.11)	0.2		
**Location**						
Masaka						
Site 1	26 (12)	13 (18)	1			
Site 2	68 (31)	20 (27)	0.64 (0.26, 1.59)			
Wakiso						
Site 1	58 (27)	22 (33)	0.98 (0.41, 2.38)			
Site 2	28 (13)	2 (3)	0.19 (0.04, 0.93)			
Mukono						
Site 1	36 (17)	13 (19)	0.90 (0.34, 2.38)	0.2		
Short travel away<1 month						
Yes vs No	99 (46)	31 (47)	1.05 (0.60, 1.84)	0.9		
***env* subtype**						
A	118 (55)	42 (63)	1			
B	0	2 (3)	-			
C	4 (6)	4 (6)	2.52 (0.49,13.0)		2.84 (0.49, 16.45)	
D	79 (37)	15 (22)	0.52 (0.27, 0.98)	0.05	0.51 (0.26, 1.00)	0.06
G	0 (0)	1 (2)	-		-	
URF	1 (0.5)	0 (0)	-		-	
***gag* subtype**						
A	97 (45)	36 (54)	1		1	
C	6 (3)	0 (0)	-		-	
D	99 (46)	21 (30)	0.53 (0.29, 0.98		0.51 (0.26, 0.97)	
G	0 (0)	3 (5)	-		-	
URF	9 (4)	9 (9)	2.57 (0.95, 6.97)	0.01	2.05 (0.68, 6.18)	0.02
**Timing of infection**						
<6 months	17 (8)	9 (13)				
6–18 months	18 (8)	5 (7)				
HIV Prevalent	181 (84)	53 (79)				
Living in household with an HIV-infected individual**						
Yes vs No	43 (20)	34 (51)	5.31 (2.98, 9.46)	<0.001	6.30 (3.40, 11.68)	<0.001

Note: categories not mutually exclusive: some clustered with different individuals in *gag* and *env*

*74 sequences from 67 individuals

**This definition includes those sequenced living with any infected person in household. Note that 44% of HIV-infected persons were sequenced (see Methods).

Of the sequences obtained, 8 of 34 (24%) clusters of HIV infections occurred within household, 59% (20/34) occurred within community and 9% cross community and the remaining 9% cross district. Non A/D subtypes were few and likely transmitted within groups limited to within community.

**Fig 5:**
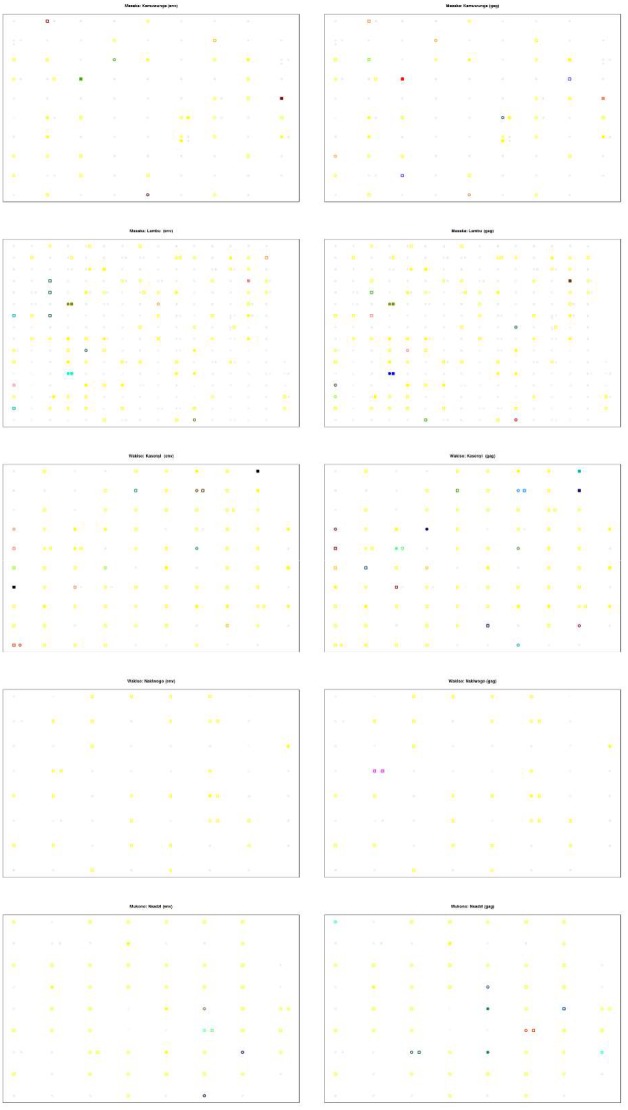
Discrete location of individuals with identified HIV clusters and gender. In all figures, the gender is indicated by shape: square = female, circle = male. Non-sequenced is indicated by grey colour, and sequenced of the non-clustered individuals is indicated by yellow. Cluster memberships are indicated by colours other than grey and yellow: the individuals here share the same colour if and only if they belong to the same cluster. Filled circles/squares are incident cases, while non-filled ones are prevalent cases. In the figure, the location of each household is randomly allocated within the corresponding village (Masaka district (Lambu, Kamuwungu), Mukono district (Nsadzi), Wakiso district (Kasenyi, Nakiwogo). Household membership, gender and prevalent or incident HIV status of the HIV infected individuals are presented by village and by gene region.

Fig 5 shows the distribution of clusters identifying sequenced and un-sequenced individuals by geographical and gene regions. Note that not all HIV infected individuals sharing a household were sequenced thus potential transmission links may have been missed. We also observed some households with >1 individual sequenced but clustering in a different household or not clustered at all. Most of the clusters tended to be within the villages. Only very few clusters crossed village borders. Within households, most infections were incident-incident transmissions, but also some prevalent individuals clustered with incident cases. Some clusters in *env* and *gag* regions were the same, but some clusters were different and some clusters occurred only in one of the gene regions. In Nakiwogo, there were no clusters in the *env* region, and only one pair occurred in the *gag* region. Also in Nsadzi, fewer clusters occurred in *gag* gene region.

### HIV phylogenetics within household partnerships

We identified 12 couples living within the same household (cohabiting couples) where both partners were HIV-infected and participated in this study. Of these couples, 7/12 (58%) clustered together, 3/7 couples that clustered included at least one incident case; 2 of these were all incident cases and one couple included a prevalent female. The remaining 5/12 (42%) couples were not linked with one another nor shared a transmission linkage with anyone else in the study.

### Identification of clusters of potential recent HIV-1 transmission

We found that 11/34 (32%) of the clusters contained at least one incident case and could be potential recent transmission clusters according to their molecular linkage and recent sero-conversion. Of the individuals in the 11 clusters (n = 24), 75% were <30 years of age, 8 were prevalent cases and 16 were incident cases. Of the prevalent individuals, 5/8 (63%) had CD4 cell count above 350 cells/mm^3^ (1 unknown) and 7/16 incident cases were early infections <6 months. The potential recent transmission clusters included 3 couples within the household (male, female), 7 clusters within village (3 male/female, 1 unique pair of females, 1 for males, 2 clusters with n = 3, male/female/female) and 1 pair of male/female cross village/community. Clusters with potential recent transmission were more likely to be younger 71% (15/21) vs 46% (21/46) and newly resident in the area 67% (14/21) vs 48% (22/46).

HIV-infected partners were identified by locating partners with respect to the incident case. Of the 11 clusters, 7 were identified where transmission could have occurred from the index HIV-positive to the initially HIV-negative, and 5 were within village, 1 was within the household and another 1 across communities. At least 5 of 7 index partners were men and reported being married but linked with partners outside their household.

**Fig 6:**
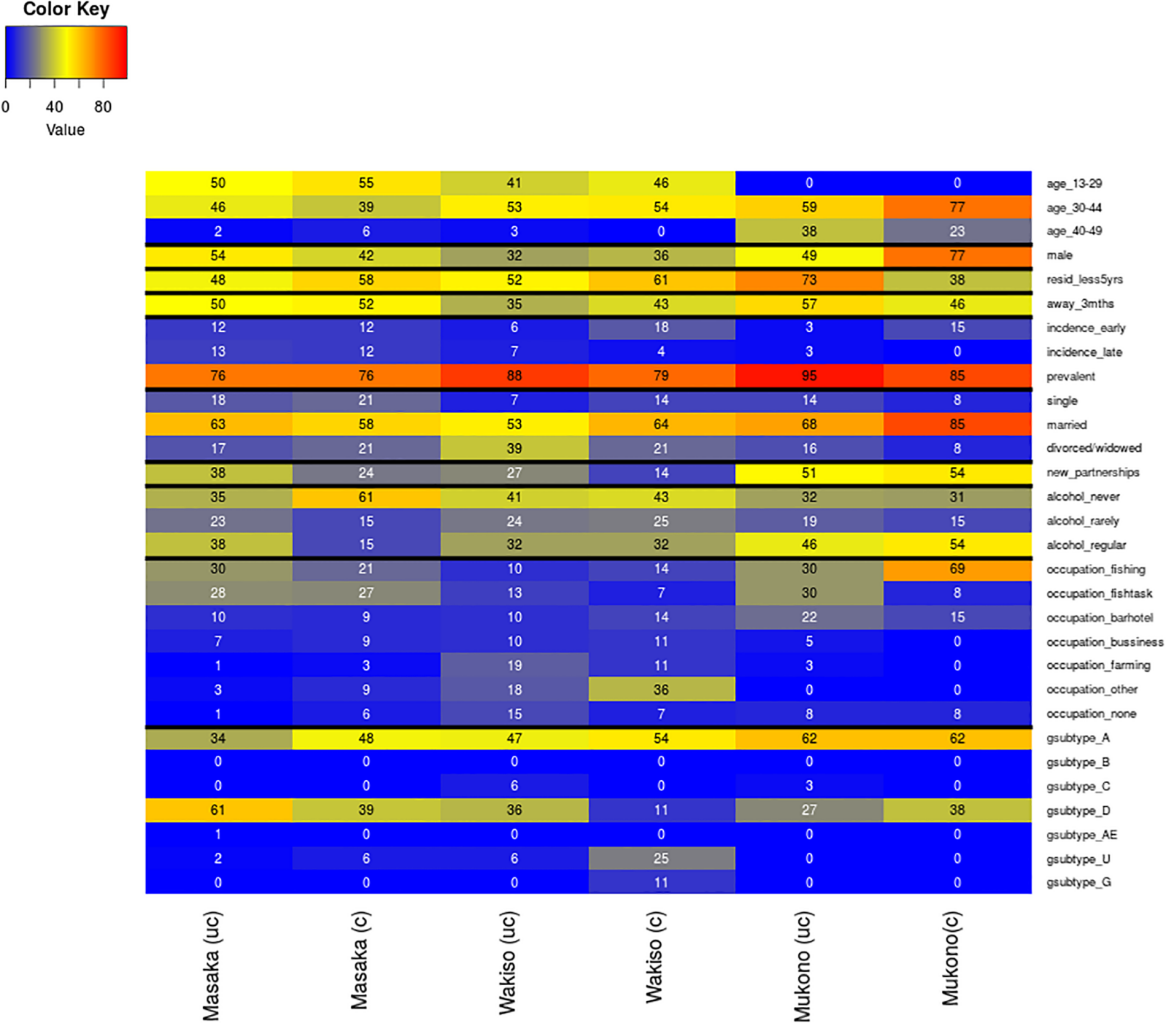
Heat map depicting clustering proportions of individuals sequenced 34 clusters (n = 283) stratified by baseline characteristics. The x-axis represents district, uc is un-clustered, c is clustered. The columns list on the y-axis are individual characteristics at study initiation and subsequent columns are the proportions clustered or un-clustered by district. Proportions are shown as a heat map to represent increasing proportions. Cells were colour coded with sliding colours as follows: blue corresponds to a low number of clustering, yellow corresponds to an intermediate amount of clustering and red corresponds to high level of clustering. Note that individuals in Masaka, Wakiso and Mukono; 2,2,1 had missing age, 25,3,1, had duration resident in area missing, 2,2,1 had missing marital status, 24,3,1 had missing occupation, 7,3,1 had alcohol use missing, 11,9,1 had partnerships missing, 7,3,1 had away 3 months missing and the remaining characteristics had none missing.

The heat map (Fig 6), shows HIV subtypes from the *gag* gene sequencing. The differences in clustering between the districts show that there is a localized component of the epidemic. In Mukono, the localized HIV transmissions are driven by the age group 30–44, males, married individuals, and individuals that have lived in the area for more than 5 years. In Wakiso and Masaka, the localized HIV transmissions are driven by married individuals and in Masaka also by the individuals that reported to never use alcohol. Other factors did not seem to be associated with clustering. Another finding highlighted was the differences in the transmitted HIV-subtypes by geographical location.

### Factors associated with transmission cluster membership

Independently, clustering was less likely in HIV-subtype D adjusted odds ratio (aOR = 0.50, 95% CI (0.26, 0.97)) and 2 times more likely in the URFs (aOR = 2.05, 95% CI (0.67, 6.18)), global p = 0.02, compared to HIV-subtype A in the *gag* region, adjusted for district location. Individuals living in a household with an HIV-infected person (any incident or prevalent) were 6 times more likely to cluster (aOR = 6.30, 95% CI (3.40, 11.68)), adjusted for *gag* subtype. Even after adjusting for district location, the associations with living with any HIV-infected person remained. Similarly considering *env* gene region, clustering was less likely in HIV-subtype D (aOR = 0.51, 95% CI (0.26, 0.99)), and ~3 times more likely in subtype-C even though less common in this setting (aOR = 2.84, 95% CI (0.49, 16.45)), compared to HIV-subtype A, global p = 0.05. There was no evidence to suggest that clustering differed by sex, age, alcohol use, short travel or marital status (p >0.2), Table 4. Subtype-C seems to circulate in groups within communities. There are 2 villages where subtype-C occurred, and 2 clusters containing subtype-C were identified; and in one cluster of 3, individuals with a transmission linkage were all <30 years.

Whilst we considered other lower epidemiologically plausible genetic distance thresholds (1.0% and 1.5%), the associations were qualitatively very similar though across the 2 more stringent genetic distance (GD) thresholds, fewer sequences were genetically linked. We obtained a total of 19 and 25 unique clusters at 1.0% and 1.5% compared to 34 clusters at 4.5%, see Table in S1 Table. Similarly, the frequency of clustering was 14% (39/283) and 18% (51/283) at 1.0% and 1.5% respectively compared to 24% at a relaxed cut-off of 4.5%. At 1.0% threshold, we had 18 pairs and 1 cluster with 3 individuals; at 1.5% cut-off clusters consisted of 24 pairs and 1 cluster of 3 individuals.

The frequency of clustering, within household, community and district remained consistent. At the more stringent thresholds, at least 80% of HIV transmission still occurred within households or within communities ie 90%, 84%, 82% at 1.0%, 1.5% and 4.5%. We observed cluster proportions of (32%, 58%, 11%, 0%) at 1%, (32%, 52%, 8%, 8%) at 1.5% versus (23.5%, 58.8%, 8.8%, 8.8%) at 4.5%, within household, community, across community and across district, respectively.

At the above GD thresholds, factors associated with clustering remained qualitatively consistent, see Table in S2 Table. Most importantly, statistically significant independent associations found between clustering and living in the same household with an HIV-infected individual remained at both cut-offs 1.0% and 1.5% in unadjusted and adjusted models. At 1.0% and 1.5% thresholds, there was insufficient evidence of associations between clustering and HIV subtype. We found none significant associations with other remaining factors age, sex, marital status, site and short travel away within 3 months.

## Discussion

Populations in the fishing communities around Lake Victoria in sub-Saharan Africa are among the hardest hit by HIV-1, yet the factors contributing to the localized epidemic are not completely understood. Combining phylogenetics and epidemiological data our study revealed 24% of individuals with sequences from the *gag*/*env* gene regions formed 34 transmission clusters. The level of clustering detected is within the 5–50% range previously estimated from several studies [16–18, 32].

We found 24% of transmission clusters occurred in partnerships within households that involve an HIV-positive partner and 59% within community. In contrast to other studies among key mobile populations showing significant HIV introductions into communities [33], it was surprising to find that most infections occurred within households and communities. In spite of the above observation, a concern remains regarding the observed >70% that were unlinked to household, other communities or districts. The role of HIV introductions into the community cannot be ruled out but could not be verified in our study. Such infections would not be prevented if interventions target the household and within community members only, thus requiring a more global interventional approach. Importantly, infections from outside households have been shown to reduce the efficacy of ART in preventing seroconversions in discordant couples [1, 34]. A general limitation of any phylogenetic approach to elucidate HIV transmission patterns is the interpretation of a definitive linkage of partners to an infecting source—third parties may be involved.

Similar to our findings, the study in Rakai district of Uganda showed that 19% of HIV positive participants with sequences from *gag* and *env* gene regions clustered among predominant HIV-subtypes A1 and D [17]. The sampling fraction in the Rakai communities was much higher and samples were collected over a longer time period, among less mobile communities compared to the fishing communities in our study. In contrast a Kenyan study of MSM and heterosexual individuals found that only 5% of sequences from the *pol* region clustered in the heterosexual cohort with HIV subtypes A1 (as predominant), B, C and D [16]. In our study clusters ranging from 2–4 individuals were identified and the majority of these were pairs. Several studies in heterosexual populations showed infrequent clustering and slower transmission dynamics among HIV non-B subtypes [35]. In many studies among non-A/D subtypes, heterosexual transmissions, show a number of transmission clusters with cluster sizes delineated into small and large clusters explained by varying populations, sampling density and cluster definitions [14, 32, 35–37].

Potential recent transmissions were associated with being younger and newly resident in the area. Of these 36% clustered with another incident suggesting high risk of HIV transmission during early infection [38]. Among HIV incident cases, the Rakai study estimated clustering within households at 39%, similar to our study [17]. Another study in Uganda (albeit a low risk population) showed that 27% sequences from incident cases in the *gag* and *env* gene regions were clustered suggesting a substantial risk of transmission in early infections in this population [18]. Findings from Rakai in Uganda [34], and a Canadian study [32] showed that some new infections could be due to sexual contacts with recently infected individuals. However, HIV clustering tends to occur among new infections and younger age group irrespective of relative transmission patterns [39]. The index partners were often male, reported being married but linked with partners outside their household suggesting that partner concurrency may contribute to a significant fraction of infections. Unless treatment as prevention is widespread at community level, interventions targeting households or discordant couples may not substantially reduce new infections in this population.

Here transmission risk in the fishing community was linked to living with an HIV-infected person, but also occurring with higher frequency within the fishing community, and more efficiently in individuals with HIV-subtype C compared to A and less likely with HIV-subtype D versus A. HIV-1 subtype-C although rare in our study population, it appears that the few individuals infected with subtype-C are transmitting HIV to their partners more efficiently compared to other subtypes. Similar associations were found within other high-risk populations [33, 40]. Furthermore, we identified a few cases where clustering occurred in only one viral region but not the other. This could be an indication of viral recombination or dual infection in a few cases [41, 42]. For example, within-household clusters, clustering in only one viral region could be due to the above reasons and subsequently masking transmission linkages. Due to the different evolutionary rates across genes, analysing more than one gene increases chances of identification of transmission clusters. Further still, recombination leads to failure to detect possible transmission networks if one gene is analysed. This is a possible explanation for why we were able to identify clusters in one gene region but failed to infer the same network in the other gene and investigations of another study yielded similar results [43]. The most ideal approach would be the generation of full HIV genomes to have a better resolution of transmission networks [44]; and if possible by next generation sequencing if analysis tools are available to resolve multiple infections.

We identified self-reported sexual partnerships within households through interviews for the demographic survey. Combining phylogenetics with the latter data provided an understanding of sexual networks at population level for example within and across communities and could identify the infecting source in some but not others due to sampling depth. The potential transmissions in younger age group suggest shorter transmission chains among younger individuals that drive HIV infections in this age group. Independently of self-reports, we observed incident-prevalent linked infections which could be prevented with ART. Whilst we did not have data on ART treatment and time of HIV infection, we cannot rule out the possibility of high HIV risk to uninfected partners indicating a failure of secondary HIV treatment as prevention. Altogether these analyses revealed a significant number of within community infections, and on the basis of discrete locator information, HIV transmission chains revealed population subgroup structures.

Data from recent studies (2011–2014) in Uganda shows that HIV incidence is still much higher among the fisherfolk community than other high risk or general population [5, 11]. In 2013 ART national guidelines included Fisherfolk as a key population eligible to receive ART at the time of diagnosis. The recent findings from START study show a benefit of early ART for all HIV-positive individuals to prevent transmissions and to address the health needs of those living with HIV [45]. On this basis, the new national ART guidelines (2016) recommend ART initiation at the earliest opportunity in all people with confirmed HIV infection regardless of CD4 cell count, and a combination HIV prevention approach including ART and safe male circumcision (SMC) has been adopted. The effectiveness of this approach remains unclear. Prior to 2013, limited access to HIV care and treatment services were available in fishing communities. The study by Kong et al in Rakai, Uganda (2007–2013) explored self-reported ART use and SMC for HIV prevention among fisherfolk and other communities [6]. They found that during mature ART/SMC scale up an SMC coverage above 40% could reduce male HIV incidence by ~39% at population level. Whereas ART coverage >20% in men was associated with lower HIV incidence at community level. Most recent data from a baseline sero-survey in 2014 from 12 fishing communities across Lake Victoria in Uganda found that SMC uptake among adults in fishing communities at baseline was between 41% and 66% (higher than 21% reported in Rakai fishing community) [46]. However, recent findings from a qualitative study on the uptake of SMC in our own study area point to continued barriers to uptake of SMC which need to be addressed if coverage is to increase [46]. While ART coverage in many fishing communities remains low (Rakai 13% in men vs 18% in women) or unknown [47]. Higher coverage of SMC and ART would greatly reduce the number of new infections.

Several limitations could influence the interpretation of our results. Due to logistical constraints, we sampled 44% of all HIV infected individuals in the cohort which meant we could not identify some clusters due to sampling, missing data and probably coverage. However in many studies these proportions are unknown, unspecified or lower than the 44% in this study [15]. The above limitation of sampling coverage may explain why we mostly identified transmission pairs and hence failing to identify the larger transmission clusters. Similar studies with higher sampling fraction within community have however still observed minimal phylogenetic clustering and high numbers of singleton lineages [17]. Whilst HIV evolutionary dynamics such as genetic distance may in part play an important role, we found that using more stringent GD thresholds did not change the associations with clustering (see above). Like many phylogenetic studies, in the absence of a fully sampled HIV transmission network, directionality of transmission cannot be ascertained but phylogenetics can identify groups that share a transmission chain [15, 48]. For example, we identified some unique male only or female only clusters, suggesting that the transmitting partner was probably not sampled. Comparison of results from several previous studies could be confounded by varying populations, individual risk profiles and methodology for clusters [15, 49, 50]. Different studies have used bootstraps ranging from 70%–99% in combination with genetic distances of 1%–4.5% [31]. Also, cluster inclusion thresholds tend to be adhoc and there is no widely accepted definition [14]. Whilst we considered several other stringent GD cut-offs, we present results for the relaxed cut-off of 4.5% as the associations with clustering were qualitatively similar. The proportion of sequences clustering ie with GD cut-off of 1.0%–4.5% was still <30%. Our results also show that a threshold of 1.5% is ideal. Other studies have shown that connections across a wide range of epidemiologically plausible GD cut-offs above GD 0.02 tended to be less informative and depended mainly on gene region, length of infection < 30 years less likely to diverge [15, 51, 52]. One should consider exploring the choice of more stringent cut-offs between 0.01 and 0.02 for example to identify potential transmission partnerships and networks particularly in settings with high-risk HIV transmission to avoid additional complexity of finding spurious associations. A large study (~20,000 sequences) that evaluated the number, size, and composition of clusters detected by Cluster Picker and HIV-TRACE at six genetic distance thresholds (1%–5.3%) on three gp41 datasets showed that the optimal gp41 genetic distance threshold to distinguish linked and unlinked couples and individuals was 5.3% and 4.0%, respectively [53]. Another potential limitation was that we were unable to estimate the proportion of HIV infections attributable to community contacts or outside community, in the absence of historical contacts. Our study shares the same inherent limitations in observational studies exploring associations with clustering. While we observed incident-incident clusters, a substantial proportion of clusters included prevalent cases. Information to look at associations of timing of infection with clustering was not available, and probably underestimated the role of new/early HIV infections. There were a number of incident cases that were unlinked but no baseline differences were found between the latter and those that were clustered. Some of the incident cases could not be linked because not all HIV infected individuals sharing a household were sampled and partners were not selectively enrolled. Despite the limitations, identifying individuals with closely related viral sequences when combined with epidemiological data is still informative for public health interventions.

In summary, to the best of our knowledge, our study reveals a first approximation to the structure of transmission chains by phylogenetics in fishing communities with high HIV prevalence and incidence, suggesting complexities in the driving forces of localized HIV transmissions. This integrated approach is needed to inform improved strategies for developing and implementing of HIV prevention programmes in fishing communities. We show that overall at least 80% of HIV transmissions continue to occur either within households or within communities suggesting localized HIV transmission even in this key mobile population. Importantly, one third of potential transmissions were incident-incident transmissions. Such individuals are unlikely to know their status which may undermine the test and treat approach. Potential recent transmissions and extra community infections in this population require more intensive HIV counseling and testing (HCT) targeting wider coverage beyond households. Though these results may well be generalizable to other high-risk fishing communities, further research to understand the contribution of recent, undiagnosed or treatment naïve infections to ongoing transmission could inform treatment as prevention strategies. These findings support a potential benefit of treatment of all positives to prevent transmissions coupled with intensified HIV counselling and testing to identify early infections.

## Supporting information

S1 Table Characteristics of clusters at genetic distance thresholds of 1% and 1.5%. (PDF)

S2 Table Logistic regression analysis for factors associated with cluster memberships at 1% and 1.5% GD thresholds. (PDF)

S1 Text Fisherfolk protocol: Risk assessment questionnaire. (PDF)

S2 Text Fisherfolk protocol: Baseline demographics questionnaire. (PDF)
