# Supplementary material for: HIV-1 transmission networks in high risk fishing communities on the shores of Lake Victoria in Uganda: A phylogenetic and epidemiological approach
Source: PLoS One. 2017 Oct 12;12(10):e0185818. doi: 10.1371/journal.pone.0185818 (PMC5638258; doi:10.1371/journal.pone.0185818)
Supplement: S1 Table — (PDF) [file pone.0185818.s001.pdf]

Characteristics of clusters at genetic distance thresholds of 1% and 1.5%

**Outcome**	**Genetic distance threshold**		
	1%	1.5%	4.5%
Proportion clustering	14%	18%	24%
Number of Clusters	19	26	34
Cluster size<br>Pairs<br>3 individuals<br>4 individuals	 18<br>1<br>0	 25<br>1<br>0	 29<br>4<br>1
Within household	31%	32%	24%
Within village/community	60%	54%	59%
Cross village/community	10%	8%	8.8%
Cross district	0%	8%	8.8%
