# Supplementary material for: HIV-1 transmission networks in high risk fishing communities on the shores of Lake Victoria in Uganda: A phylogenetic and epidemiological approach
Source: PLoS One. 2017 Oct 12;12(10):e0185818. doi: 10.1371/journal.pone.0185818 (PMC5638258; doi:10.1371/journal.pone.0185818)
Supplement: S2 Table — (PDF) [file pone.0185818.s002.pdf]

Logistic regression analysis for factors associated with cluster memberships at 1% and 1.5% GD thresholds

**GD threshold**	**1.5%***	**1.5%***	**1%***	**1%***
Factor	Unadjusted OR	Adjusted OR	Unadjusted OR	Adjusted OR
Sex: Male	0.90 (0.48, 1.69)		1.08 (0.53, 2.17)	
Age<br>13-29<br>30-44<br>45-50	 1<br>0.93 (0.45, 1.76)<br>0.76 (0.09,6.56)		 1<br>1.08 (0.54, 2.18)<br>-	
Marital status				
Single	1		1	
Married	1.20 (0.45, 3.19)		1.05 (0.36, 3.02)	
Divorced	0.80 (0.23,2.75)		0.63 (0.15, 2.54)	
Widowed	0.71 (0.23, 2.20)		0.67 (0.19, 2.28)	
Alcohol use<br>Never<br>Rarely<br>Regularly	 1<br>0.72 (0.32, 1.62)<br>0.69 (0.34, 1.41)		 1<br>0.91 (0.36, 2.29)<br>0.97 (0.44, 2.15)	
Short travel away <1 month<br>Yes vs no	1.14 (0.61, 2.13)		1.45 (0.72, 2.94)	
Env<br>A<br>B<br>C<br>D<br>G<br>URF	 1<br>-<br>4.27 (0.82, 22.19)<br>0.64 (0.31, 1.32)<br>-<br>-	 <br>-<br>6.04 (1.41, 5.62)<br>0.65 (1.13, 32.2)<br>-<br>-	 1<br>-<br>5.54 (1.06, 29.10)<br>0.39 (0.15, 0.98)<br>-<br>-	
Gag<br>A vs D	1.01 (0.53, 1.92)		0.76 (0.37, 1.56)	
Timing of infection				
Living in the same household with an HIV-infected individual	2.74 (1.42, 5.28)	2.82 (1.41, 5.62)	3.09 (1.49, 6.39)	2.89 (1.39, 6.04)

*Data using GD threshold 1%, 1.5%, unique clusters (4 clustered in both gag and env) from 283 study participants.
