# Supplementary material for: HIV-1 transmission networks in high risk fishing communities on the shores of Lake Victoria in Uganda: A phylogenetic and epidemiological approach
Source: PLoS One. 2017 Oct 12;12(10):e0185818. doi: 10.1371/journal.pone.0185818 (PMC5638258; doi:10.1371/journal.pone.0185818)
Supplement: S1 Text — (PDF) [file pone.0185818.s003.pdf]

FISHERFOLK PROTOCOL: Risk Assessment Questionnaire (RAQ)

Visit Code	|__|__|.|__|[VISCODE]
Volunteer ID Number	|__|__|__|__|__|__|__|__|[VIN]
Date of Visit (DD-MMM-YYYY)	|__|__|-|__|__|__|-|__|__|__|__| [VISDATE]

1. During the last 3 months, on average, how often have you taken alcohol?	*Select one* [ALCHOL]<br>☐ Never<br>☐ Dailv<br>☐ Weekly<br>☐ Monthly<br>☐ Less than Monthly
2. Some people have tried injecting illicit drugs using a needle and syringe. In the last 3 months, have you injected any illicit drugs?	*Select one* [INJDR]<br>☐ Yes<br>☐ No<br>☐ I don't Know<br>☐ Refused to answer
3. Some people have tried a range of different types of drugs. In the last 3 months which of the following, if any, have you used?)	*Select one response for each*<br>Yes / No / Don't Know
3a. Mairungi (Khat / Miraa)	☐ ☐ ☐ [KHAT]
3b. Glue/Petrol sniffing	☐ ☐ ☐ [PGLUE]
3c. Enjaga (Marijuana)	☐ ☐ ☐ [ENJAG]
3d.Other, specify [OTHSP]	☐ ☐ ☐ [OTHER]
4. In the last three months, have you had any abnormal or Smelly genital or urethral discharge?	*Select one*<br>☐Yes ☐No ☐ Don't Know<br>[GENDIS]
5. In the last three months, have you had genital sores or Ulcers?	*Select one*<br>☐Yes ☐No ☐Don't Know<br>[GENSORE]

6. If you said yes to questions 4 and 5, did you seek treatment for it? |__|Yes |__| No |__|Not asked [TREAT]

7. In the last 3 months have you had a doctor or nurse tell you that you had an STI?|__|Yes |__| No [STI]

Visit Code	|__|__|.|__| [VISCODE]
Volunteer ID Number	|__|__|__|__|__|__|__| [VIN]
Date of Visit (DD-MMM-YYYY)	|__|__|-|__|__|__|-|__|__|__|__| [VISDATE]

Speak this aloud to the volunteer:

(Speak this aloud to the volunteer: Now we would like to ask you about your sexual partners and Condom use. Some of these questions may be sensitive or uncomfortable to you, but everyone is asked the same questions. Some questions may not be relevant to you. Remember that all answers you give are confidential. It is very important for this research study that you give honest answers. ***When we talk about sex, we mean when you put your penis into a partner's vagina or a partner puts his penis into your vagina)***

8. In the last three months, how many sex partners have you had?<br><br>*(If 00 in the Last three Months Go to Q9(a))*	*Enter number* |__|__| <br>*Enter 99 if there were too many to remember)* [PSEX]
8a. Of these ____(say the total number of sex partners given in Q8) sex partners, how many were <u>new partners</u>, that is, Someone you never had sex with before?)<br><br>*(If no new partner skip to Q9a)*	*Enter number* |__|__| [NPSEX]<br>99 if there were too many to remember
8b. How frequently did you use condoms when having sex with these (repeat/say number in 8a) new sex partners?<br>***(Ask for each new partner probe condom use and reason for use)***	*Select one*<br>Codes for condom use:<br>1=Never<br>2=Sometimes(Less than half the time)<br>3=Frequently (More than half the time)<br>4=Always

New Partner	Condom Use (Codes)	Reason
NP1	Codes ? [CONDU1]	[R1]
NP2	[CONDU2]	[R2]
NP3	[CONDU3]	[R3]
NP4	[CONDU4]	[R4]

9a. Do you know if any of your sex partners is HIV infected?<br><br>*(If Q9a is No then go to Q10)*	*Select one*<br>☐ Yes<br>☐ No<br><br>[PARTSTAT]

Visit Code	|__|__|.|__| [VISCODE]
Volunteer ID Number	|__|__|__|__|__|__|__| [VIN]
Date of Visit (DD-MMM-YYYY)	|__|__|-|__|__|__|-|__|__|__|__| [VISDATE]

9b. How frequently did you use condoms when having sex with this/these *(Q.9a)* HIV infected partner/s?	***Select one***<br>☐ Never<br>☐ Sometimes (less than half the time)<br>☐ Frequently- (more than half the time)<br>☐ Always<br>[CUSEHIV]
9c. Do you know if any of your HIV infected partners is on Antiretroviral therapy? ***(If No/skip to Q.10)***	***Enter code in the box on the right***<br>1=Yes ☐<br>2=No<br>[ART]
9d. How frequently did you use condoms when having sex with these HIV infected partner/s on Antiretroviral therapy?	***Select one***<br>☐ Never<br>☐ Sometimes –(less than half the time)<br>☐ Frequently- (more than half the time)<br>☐ Always [CART]
10.Of these __(say the sum of the total number of sex Partners given inQ8 and Q9) sex partners, how many have you had sex with even one time while under the influence of alcohol or drugs?	***Enter number*** |__|__| [DSEX]<br>***(99 if there were too many to Remember)***

Visit Code	|__|__|.|__| [VISCODE]
Volunteer ID Number	|__|__|__|__|__|__|__|
Date of Visit (DD-MMM-YYYY)	|__|__|-|__|__|__|-|__|__|__|__| [VISDATE]

11.<br>Of these ______ (say the sum of the total number of sex partners given in Q8 and Q9) sex partners, how many did you receive any money, gifts, or help from in exchange for having sex with them? By gifts or help I mean, buying clothes, alcohol, food Paying rent, money or anything else.<br>Payment may or may not have been at the same time as the Sex.	***Enter number*** |__|__| ***[Enter 99 if there were too many to remember]***<br>[GIFTR]
12.<br>Of these ______ (say the sum of the total number of sex partners given in Q8and Q9) sex partners, how many did you give any money, gifts, or help to in exchange for having sex with them? By gifts or help I mean, buying clothes, alcohol, food, paying rent, transport, money or anything else.<br>Payment may or may not have been at the same time as the sex.	***Enter number*** |__|__| ***(Enter 99 if there were too many to remember)***<br>[GIFTG]
13. In the last three months, have you been forced to have sex against your will?<br>*If yes or refused to answer send for additional counseling services*	[FSEX]<br>☐ Yes ☐ No ☐ Refused to answer<br>***Select one***
14. In the past month, have you travelled or stayed away from home frequently? (More than two nights on average in a month)	☐ Yes ☐ No<br>[HAWAY]

Please Initial and date the appropriate section below

Filled by: ____________ _____/_____/_________
Initials Date

Reviewed by: ____________ _____/_____/_________
Initials Date
