# Supplementary material for: HIV-1 transmission networks in high risk fishing communities on the shores of Lake Victoria in Uganda: A phylogenetic and epidemiological approach
Source: PLoS One. 2017 Oct 12;12(10):e0185818. doi: 10.1371/journal.pone.0185818 (PMC5638258; doi:10.1371/journal.pone.0185818)
Supplement: S2 Text — (PDF) [file pone.0185818.s004.pdf]

Volunteer ID Number[VIN]

☐☐☐☐☐☐☐

Visit Date[VISDATE]

☐☐ ☐☐☐ ☐☐☐☐

Refer to study CRF Instructions to complete this form

1. What is your tribal or ethnic group? [ETHNGRP] ________________

2. What is your country of birth? [BCTRY] ________________

3. How many total years of school have you completed in each category? *(Enter '00' if no education in that category)*

3a. Primary ☐☐ [YRPRIM]

3b. Secondary: ☐☐ [YRSEC]

3c. Post-secondary: ☐☐ [YRPSEC]

3d. Completed years of other school or apprenticeship: ☐☐ [YROTH]

3e. If Q3d is not '00', specify type of other school/training below: [OTHSCH]

____

4. What is your religion? *(mark one only)* [RELIGON]

☐ Catholic ☐ Muslim

☐ Protestant ☐ None

☐ Other Christian ☐ Other, specify: ________________

5. How long have you lived in this area? *(Enter duration in months and 00 if less than one month)*

☐☐ Months [DURLIVED]

6. What is your current marital status? [MARITAL]

☐ Single *(never married)* ☐ Married, monogamous ☐ Widowed

☐ Divorced/separated ☐ Married, polygamous

7. What has been your primary source of income in the last 12 months? *(Mark up to two choices)*

☐ Selling fish	[SELLF]	☐ Bar	[BAR]	☐ Loading	[LOAD]
☐ Boat hire	[BHIRE]	☐ Cleaning Nets	[CNETS]	☐ Fish Processing	[FISHP]
☐ Transporting	[TRNS]	☐ Farming	[FARM]	☐ Fishing	[FISH]
☐ House Rent	[RENT]	☐ Other, specify:	[7OTHSP] ________________		

8. Are you currently caring (financially) for any dependants? [DEP]

☐ Yes ☐ No

9. If yes to Q8, how many dependants do you have? [DEPNO]

☐☐ (Enter No of Dependants)

Please Initial and date the appropriate section below:

Filled by: ________ ____/________/______________

Initials Date

Reviewed by:_______ _____/_______/____________

Initials Date
